# Developmental Dysplasia of the Hip and Laterality: The Importance of Graded Severity of the Contralateral Hip

**DOI:** 10.1007/s43465-024-01157-9

**Published:** 2024-07-06

**Authors:** Emily K. Schaeffer, Charles T. Price, Kishore Mulpuri, Global Hip Dysplasia (GHD) Study Group

**Affiliations:** 1 Department of Orthopaedic Surgery, British Columbia Children’s Hospital, Vancouver, BC Canada; 2 Department of Orthopaedics, University of British Columbia, Vancouver, BC Canada; 3 Arnold Palmer Medical Center, Orlando, FL USA

**Keywords:** Developmental dysplasia of the hip, DDH, Laterality, Bilateral, Hip dysplasia, Hip instability, Hip dislocation, Graded severity

## Abstract

**Background:**

Laterality and bilaterality have been reported as prognostic variables in developmental dysplasia of the hip (DDH) outcomes. However, there is little clarity across the literature on the reporting of laterality in developmental dysplasia of the hip (DDH) due to the variability in severity of the condition. It is widely accepted that the left hip is most frequently affected; however, the true incidence of unilateral left, unilateral right and bilateral cases can be hard to quantify and compare across studies. The purpose of this study was to examine laterality accounting for graded severity in a multi-centre, international prospective observational study of infants with hip dysplasia to demonstrate the complexity of this issue.

**Methods:**

A multi-centre, prospective hip dysplasia database was analyzed from 2010 to April 2015. Baseline diagnosis was used to classify patients into a graded laterality category accounting for hip status within the DDH spectrum.

**Results:**

A total of 496 patients were included in the analysis; 328 were <6 months old at diagnosis and 168 were between 6 and 18 months old. Of these patients, 421 had at least one frankly dislocated hip. Unilateral left hip dislocations were most common, with 223 patients, followed by unilateral right and bilateral dislocations with 106 and 92 respectively. Stratifying these patients based on status of the contralateral hip, 54 unilateral left and 31 unilateral right dislocated patients also had a dysplastic or unstable contralateral hip. There were significantly fewer bilateral patients in the 6 to 18-month group (*p* = 0.0005). When classifying laterality by affected hip, bilaterality became the predominant finding, comprising 42% of all patients.

**Conclusions:**

Findings from this multi-centre prospective study demonstrate the necessity to account for the graded severity in hip status when reporting DDH laterality. To accurately compare laterality across studies, a standardized, comprehensive classification should be established, as contralateral hip status may impact prognosis and treatment outcomes.

**Level of Evidence:**

Level II Prognostic Study.

## Introduction

Developmental dysplasia of the hip (DDH) describes a spectrum of hip disorders ranging from mild instability to a complete dislocation. DDH is the most common pediatric hip disorder, with 1–3% of all newborns being diagnosed at birth [1, 2], and approximately 80% of cases are female [3, 4]. The status of the contralateral hip may influence clinical and patient outcomes, guide or alter treatment approaches and impact overall long-term prognosis. Several studies have commented on bilateral dislocations being more difficult to treat by conservative management [5, 6], while others have refuted that finding [7, 8]. Consequently, an accurate understanding of laterality in DDH is paramount to guiding these clinical decisions. Historically, hip dysplasia is considered to most frequently manifest as a pathology of the left hip, followed by bilateral involvement, while a unilateral right hip pathology is least frequently observed [9]. The left hip is thought to be most commonly affected because of intrauterine positioning. Greater stress is placed on the left hip when a baby is in a normal position in the womb, restricting abduction and promoting adduction.

Examining laterality and directly comparing the frequency of left and right affected hips is a complex issue, particularly in DDH. The complexity arises from the fact that DDH spans such a broad spectrum of hip joint disorders, from mild dysplasia to a completely dislocated hip [10–15]. Therefore, there is discrepancy and ambiguity in the literature when cases are reported as unilateral and bilateral. Additionally, consistency across studies in the literature is often difficult because some studies involve purely dislocated hips [16], while some involve dysplastic hips [17] and others involve a combination or complete spectrum of the disorder [18–20]. The purpose of this study is to report bilaterality in the various forms ranging from dislocation to mild dysplasia to effectively classify a hip condition that involves graded laterality.

## Materials and Methods

The International Hip Dysplasia Institute (IHDI), established in 2008, is a not-for-profit consortium of medical and research professionals and benefactors who strive to improve the health and quality of life for individuals and families affected by DDH through advocacy, education, and collaborative research [21]. In 2010, IHDI began a prospective, multi-centre, observational cohort study of children younger than 18 months old with a diagnosis of DDH. At the time, this study was the largest of its scope that prospectively collected diagnosis, treatment, and outcome data on DDH in infants. As such, the study was able to uniquely examine laterality and the respective frequencies of left, right, and bilateral DDH and compare to the existing literature. According to the IHDI study criteria, infants younger than 6 months old were included and enrolled only if at least one hip was frankly dislocated. Those with dislocatable, subluxable, or dysplastic hips in the absence of a dislocation were excluded. However, these forms of DDH were recorded if contralateral to a frankly dislocated hip. Infants older than 6 months were eligible for the study with any form of DDH.

The IHDI study database was analyzed from 2010 to April 2015. Data were collected and managed using REDCap electronic data capture tools. Baseline diagnosis was determined across all centres. All diagnoses were confirmed by ultrasound or radiograph prior to brace or surgical treatment. Children less than 6 months of age at initial diagnosis were included if at least one hip was frankly dislocated, while children 6–18 months of age were included if at least one hip had some form of hip dysplasia. Patients with known or suspected neuromuscular, collagen, chromosomal or lower extremity congenital anomalies, or patients <6 months of age that did not have a frankly dislocated hip were excluded from the study. Patients who previously received treatment with orthosis or surgery were also excluded.

Basic descriptive statistics were used on baseline diagnoses. Chi square analysis was performed to determine differences between diagnostic categories and age groups.

## Results

### Dislocated Hip Laterality

In total, 496 patients were included in analysis; 328 were <6 months of age at diagnosis and 168 were 6–18 months. Of the included patients, 421 had at least one frankly dislocated hip (Table 1) and 75 patients had dysplastic or unstable hips with no dislocation (Table 2). Unilateral left dislocations were most common, with 223 patients, followed by unilateral right (106) then bilateral (92). There were significantly fewer bilateral cases in the older compared to the younger age group (*p* = 0.0005).

**Table 1:** Dislocated hip laterality

Dislocated laterality	<6 months	6–18 months	Total
Unilateral right	76	30	106
Unilateral left	167	56	223
Bilateral	85	7	92
total	328	93	421

**Table 2:** Dysplastic Hip laterality (no dislocations)

Dysplastic laterality	6–18 months
Unilateral right	4
Unilateral left	7
Bilateral	64
Total	75

We next stratified these patients by the status of the contralateral hip (Table 3). Of the 223 unilateral left dislocated patients, 54 had a dysplastic or unstable right hip, while 31/106 unilateral right dislocated patients had a dysplastic or unstable left hip. The contralateral hip was affected significantly less in older patients, with only 3/86 (*p* < 0.00001).

**Table 3:** Dislocated hip laterality including contralateral hip status

Dislocation status	Contralateral hip	<6 months	6–18 months	Total
Unilateral right	Reduced	47	28	75
	Affected	29	2	31
Unilateral left	Reduced	114	55	169
	Affected	53	1	54
Bilateral	Dislocated	85	7	92
Total		328	93	421

### Affected Hip Laterality

Continuing analysis of the 421 patients with at least one dislocated hip, we next examined affected hip laterality. A patient was considered bilateral if the hip contralateral to the dislocation was dysplastic, dislocatable or subluxable. Bilateral cases were predominant (177 cases), followed by unilateral left (169), then unilateral right (75) (Table 4).

**Table 4:** Affected laterality of patients with at least one dislocated hip

Affected laterality (all dislocated patients)	<6 months	6–18 months	Total
Unilateral right	47	28	75
Unilateral left	114	55	169
Bilateral	167	10	177
Total	328	93	421

Finally, when including patients with dysplastic or unstable hips only, bilateral cases predominated in each age group, and across the study with 241 patients, followed by 176 unilateral left and 79 unilateral right patients (Table 5).

**Table 5:** Affected laterality of all patients (dislocated and dysplastic)

Affected laterality (all patients)	<6 months	6–18 months	Total
Unilateral right	47	32	79
Unilateral left	114	62	176
Bilateral	167	74	241
Total	328	168	496

## Discussion

We can consider laterality in a number of different ways both in regard to the DDH spectrum and within the context of this study. First, when considering laterality from a dislocated hip perspective, a case would only be designated as bilateral if both hips are frankly dislocated. A unilateral designation would, therefore, be ascribed to any patient with a single dislocated hip, regardless of the presence or absence of instability in the contralateral hip. Second, we can consider laterality by gradation. In this instance, there are five distinct categories: (1) unilateral left dislocation with a reduced right hip; (2) unilateral left dislocation with a dysplastic/unstable right hip; (3) unilateral right dislocation with a reduced left hip; (4) unilateral right dislocation with a dysplastic/unstable left hip; (5) bilateral dislocation. Third, by looking at dysplasia separately from dislocations, a unilateral left patient can have a dislocated or dyplastic/unstable left hip; a unilateral right patient can have a dislocated or dysplastic/unstable right hip; and a bilateral patient can have 2 dislocated hips or 2 dysplastic/unstable hips. Finally, we can also consider any affected hip to count towards a patient’s laterality. In that case, a unilateral left case would be constituted by a dislocated, dysplastic, dislocatable or subluxable left hip with a reduced and stable right hip; a unilateral right case would be constituted by a dislocated, dysplastic, dislocatable or subluxable right hip with a reduced and stable left hip; and a bilateral case would be constituted by any combination of dislocated, dysplastic, dislocatable, subluxable right and left hips. A seemingly simple classification system assigning a patient as unilateral left, unilateral right or bilateral rapidly becomes an issue rife with complexities and subjective opinions. The literature does not necessarily provide clarity as to a standardized method for reporting patient laterality in DDH. Additionally, even if laterality is well-defined within the context of a specific DDH study, that study’s definition of laterality does not necessarily align with another study. Despite this, authors have long been equating what the “literature” states for the overall frequency of left and right affected hips, generalizing across published works.

The inherent issue rooted in this practice becomes apparent when examining laterality frequency of patients enrolled in this multi-centre IHDI study. In total, 496 patients were included in analysis. Of those patients, 328 were less than 6 months old at the time of diagnosis, and 168 were 6–18 months old at diagnosis. Due to the study inclusion criteria, all patients less than 6 months had at least one frankly dislocated hip; however, contralateral dysplasia, subluxation or other instability was not precluded, and was recorded. Of the 168 patients diagnosed after the age of 6 months, 93 had at least one frankly dislocated hip, while 75 had no dislocation, but did have some form of hip dysplasia.

In total, 421 patients included in analysis had at least one frankly dislocated hip (Table 1), while 75 patients had dysplastic or unstable hips (Table 2). The left hip was most commonly dislocated in both the younger and older age groups, with 223 total patients with a unilateral left dislocation (Table 1). This finding is in line with the historical literature, and expected due to the excess stress placed on the left hip in utero. However, there were more instances of unilateral right dislocations than bilateral dislocations, with 106 and 92 cases, respectively. These findings are not out of the realm of the range found in the literature, as some sources state the prevalence of unilateral right and bilateral cases to be approximately equal [9]. Stratifying for age group, patients <6 months old at diagnosis more closely followed the traditionally reported frequency, with 76 unilateral right, 167 unilateral left and 85 bilateral cases. In contrast, patients older than 6 months at diagnosis had surprisingly few cases of bilaterality in comparison, with 7 bilateral patients, 30 unilateral right and 56 unilateral left. It has been posited that bilateral cases could potentially be expected to be more frequent in older patients—those with late-presenting DDH—due to increased difficulty in diagnosing symmetrical dislocations. Specifically, Haasbeek et al. reported a predominance of bilateral cases in late-diagnosed patients [22]. They found a 28% incidence in early-diagnosed and 44% incidence in late diagnosed [22]. The findings of our study however, appear to refute this, with significantly less bilateral cases than would be expected from the total study population (*p* = 0.0005). These findings suggest an age-dependent laterality frequency that is not necessarily accounted for when generalizing across the literature. The discrepancy between these two studies may potentially be explained by a late diagnosis in the Hassbeek study being defined as at or after 20 months of age.

We next examined this same study population, stratifying patients with at least one dislocated hip according to the status of the contralateral hip (Table 3). We found a relatively substantial number of patients with a unilateral dislocation that also have some degree of dysplasia or instability in their contralateral hip. In 29% of unilateral right dislocations, the left hip was either dysplastic, dislocatable or subluxable; likewise of the right hip in 24% of unilateral left dislocations. Notably, an affected contralateral hip was seen significantly less in older patients, representing only 6.7 and 1.8% of unilateral right and left cases, respectively (*p* < 0.00001). This observation may possibly be due to a tendency for milder forms of hip instability to spontaneously resolve as the infant matures.

Treatment strategies, management, and ultimately patient outcome, may be influenced by the status of the contralateral hip, but without a standardized method of reporting this across studies in the literature, how can we begin to compare and evaluate these outcomes? The Pavlik harness is the predominant first line treatment in infants diagnosed prior to 6 months of age, and in older infants with hip instability or dysplasia, but not a complete dislocation. This raises several questions with regard to this management choice. In unilateral cases, is Pavlik harness treatment success affected by a dysplastic or unstable contralateral hip compared to a reduced hip? How does bracing success compare between a patient with a bilateral dislocation and a patient with a unilateral dislocation and a subluxable contralateral hip? Are success rates comparable, and if not, are the differences meaningful from a clinical and patient perspective?

Currently, we do not have an accurate and complete understanding of the impact of graded laterality in hip dysplasia, and the multitude of ways available to report laterality only compounds the issue. Once again examining our study population of all infants with at least one dislocated hip, we next considered a case to be bilateral if the hip contralateral to the dislocation was affected (dysplastic, subluxable or dislocatable). This resulted in the number of bilateral cases accounting for 50.9% of all cases in the younger age group and being the most frequent observance across the whole study (42%) (Table 4). In the older age group though, again, the prevalence of bilateral cases was significantly lower compared to unilateral cases and compared to bilateral cases in the younger age group (*p* < 0.00001). Considering the entire population of patients with at least one dislocated hip, however, bilaterality becomes the most common occurrence when including the status of the non-dislocated contralateral hip. This is consistent with the findings of a study that looked at the frequency of irregular morphology of the contralateral hip in adult patients with hip pain secondary to DDH. However, this study did not stratify patients across the spectrum of DDH, and instead included only dysplastic or subluxed hips that had gone untreated during infancy [17].

Finally, we extended our analysis to all 496 patients in the study, including those 6–18 months with dysplastic or dislocatable/subluxable hips only. Given the large number of bilateral dysplastic cases (64/75) (Table 2), the total number of bilateral cases (any combination of dislocated, dysplastic, dislocatable or subluxable hips) becomes the largest group of patients in each individual age group, and across the study with 241 patients total (48.6% of the study population) (Table 5).

One limitation to this study is that patients <6 months old with dysplastic or unstable hips in the absence of dislocation were not included. Additionally, Lee et al. show that some cases of mild dysplasia seem to present later in life [23]. However, the study suggests that adolescent/adult dysplasia may be a distinct pathology from DDH and would thus be considered separately. It is also possible that contralateral dysplasia/instability may be over diagnosed in this particular cohort. Given that most children were diagnosed at or beyond 6 weeks of age with prominently dislocated hips, the implementation of brace treatment was introduced. As a result, this may have hindered the ability to determine whether certain contralateral hips may have self-resolved with time. However, the purpose of this study wasn’t to define the true incidence of contralateral dysplasia or instability, but rather to illustrate the concept of graded severity. Another limitation to the study is that, although we propose a more comprehensive laterality classification system, there still remains unaddressed complexities. Specifically, even in a dislocated hip there is a range of severity in both extent of dislocation and reducibility. This severity varied across centres involved in the study, as did patient recruitment. Finally, extensive statistical testing was not performed as this was intended as a thought-generating study about the need for clarity in laterality classification.

**Fig. 1:**
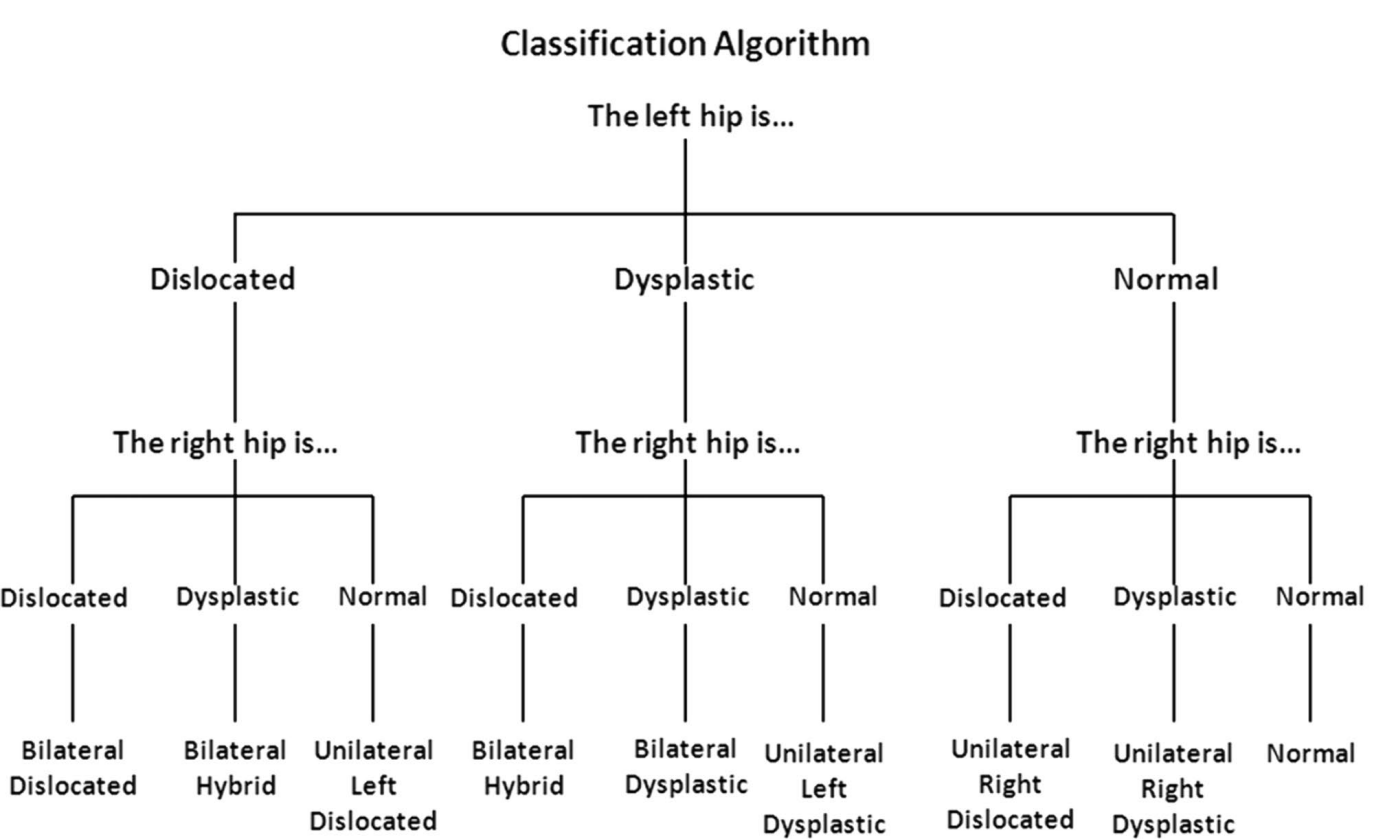
Laterality classification algorithm. Suggested methodology and classification system for determining the laterality of a patient with DDH to standardize and more comprehensively span the DDH spectrum

This study presents the complexity of issues surrounding the reporting of laterality in hip dysplasia. Within this study population alone, we have four distinct ways of presenting and discussing hip laterality in regard to DDH. Further, each of these methods produces a distinct distribution of unilateral left, unilateral right, and bilateral patients. What is the impact of each of these different ways of reporting laterality? Without adequate specification across the literature, how can we be certain when reporting whether a laterality trend is consistent with the existing evidence? Additionally, we do not yet know the relevancy of each of these reporting methods. When dealing with dislocated hips, perhaps only the dislocation status is necessary to provide prognostic or treatment information for that particular patient. Concerted effort amongst the orthopaedic surgery community to classify laterality accurately and comprehensively may be necessary to facilitate cross-study comparisons. Until then, the true incidence of unilateral versus bilateral cases is difficult to extrapolate from the literature. To provide clarity, the authors suggest a classification system (Table 6) and algorithm (Fig. 1) based on data from the International Hip Dysplasia Institute that encompasses the DDH spectrum: unilateral dislocated, unilateral dysplastic (left/right), bilateral dislocated, bilateral dysplastic or bilateral hybrid (dysplastic-dislocated). We have since adopted this approach to classifying laterality in the transition of this study to the current Global Hip Dysplasia Registry (GHDR). Standardized reporting may then aid the discovery of specific treatment outcomes and prognostic indicator patterns based on affected hips.

**Table 6:** Laterality classification system

Classification	Hip status	
	Right	Left
Unilateral dislocated	Dislocated	Normal
	Normal	Dislocated
Bilateral dislocated	Dislocated	Dislocated
Unilateral dysplastic	Dysplastic	Normal
	Normal	Dysplastic
Bilateral dysplastic	Dysplastic	Dysplastic
Bilateral hybrid	Dislocated	Dysplastic
	Dysplastic	Dislocated
